# Impact of the COVID-19 Pandemic on the Usage of Blood for Transfusions: A 2-Year Experience from a Tertiary Center in Korea

**DOI:** 10.3390/vaccines11030585

**Published:** 2023-03-03

**Authors:** Juhye Roh, Jangwook Lee, Jinyoung Park, Hee Jung Kang, Young Kyung Lee, Han-Sung Kim, Yonggeun Cho

**Affiliations:** 1 Department of Laboratory Medicine, Hallym University Sacred Heart Hospital, Anyang 14068, Republic of Korea; 2 Department of Internal Medicine, Dongguk University Ilsan Hospital, Dongguk University College of Medicine, Goyang 10326, Republic of Korea; 3 Department of Psychology and Neuroscience, Duke University, Durham, NC 27708, USA

**Keywords:** COVID-19, blood supply, blood shortage, blood usage, transfusion rate, postoperative transfusion

## Abstract

The coronavirus disease (COVID-19) outbreak affected the utilization and management of blood products in hospitals. Blood shortages occurred owing to social distancing policies and reduction in blood donors. However, only a few studies examined whether these changes affected blood usage and transfusion patterns. We retrospectively reviewed blood component usage according to hospital departments and phases of surgery in transfused patients admitted between 1 March 2019 and 28 February 2021, in a single center in Anyang, Korea. We also analyzed the length of hospital stay and mortality to determine prognosis. In 2020, 32,050 blood components were transfused to 2877 patients, corresponding to 15.8% and 11.8% less than the rates in 2019, respectively. Postoperative usage of blood products significantly decreased in 2020 (3.87 ± 6.50) compared to 2019 (7.12 ± 21.71) (*p* = 0.047). The length of hospital stay of the patients who underwent postoperative transfusion in 2019 (*n* = 197) was 13.97 ± 11.95 days, which was not significantly different from that in 2020 (*n* = 167), i.e., 16.44 ± 17.90 days (*p* = 0.118). Further, 9 of 197 postoperative transfusion patients died in 2019, while 8 of 167 patients died in 2020 (*p* = 0.920). The COVID-19 pandemic resulted in limited blood supply and reduced postoperative transfusions; however, patient prognosis was not affected.

## 1. Introduction

The continuing coronavirus disease (COVID-19) pandemic has become a major global concern. It was challenging to find an appropriate therapy at the beginning of the pandemic in the context of the COVID-19 impact on clinical pathways, and this caused a delay in providing appropriate treatment [1]. The unprecedented increase in the infectious disease burden due to COVID-19 has affected hospital policies on the utilization of finite resources for patient care [2,3]. The pattern of patients’ hospital utilization has also changed due to concerns about the pandemic and social distancing campaigns [4]. While there was an apparent reduction in the overall number of patients visiting the hospital [5,6], the prioritization of medical personnel and facilities for screening, isolation, and treatment of patients with COVID-19 could have led to increased numbers of patients with other diseases in the hospitals. These opposing factors resulted in an overall increase in the length of hospital stay and reduced access to the hospital during the pandemic era [7,8,9].

The aspect of blood transfusion might have suffered most conspicuously from the social distancing policy and people’s reluctance to visit healthcare centers, as reflected by the drastic decrease in healthy blood donors visiting the blood donation centers [10,11,12,13]. In addition to the reduction in the number of visitors, precautionary actions of many blood collection facilities, such as exclusion or deferral of high-risk donors, further reduced the supply of blood products to hospitals [14]. Confronted with the chronic deficit in the supply of blood products, blood banks in the hospitals had to restrict the release of blood units based on established rationales for patient blood management [15,16].

However, only a few studies have examined whether the changing environment of blood banks due to the COVID-19 pandemic affected blood usage and transfusion patterns. Therefore, we aimed to systematically analyze the changes in blood consumption and determine which factors were predominantly affected by the pandemic. Furthermore, the prognosis of the patients was also analyzed to determine how these changes affected patient outcomes.

## 2. Materials and Methods

### 2.1. Study Design

We performed a retrospective descriptive study of 2-year data. The data were obtained from a blood bank supporting an 830-bedded tertiary care academic hospital in Anyang, South Korea. To assess the impact of the COVID-19 pandemic on the blood usage patterns of the hospital, the period from March 2020 to February 2021 was set as the study period—“year 2020.” The study period was compared with the same period in the previous year, i.e., from March 2019 to February 2020, which was set as the control period—“year 2019.” All the patients who received blood transfusion during the study period were eligible for inclusion. The study was approved by the Institutional Review Board (IRB) of the Hallym University Sacred Heart Hospital (IRB No. 2021-06-018).

### 2.2. Data Collection and Analysis

The records of all transfused patients admitted to our hospital between 1 March 2019 and 28 February 2021 were reviewed. Data were extracted from the Hallym Smart Clinical Data Warehouse, which is a longitudinal de-identified database of patients admitted to the Hallym University Sacred Hospital established in 2011. The following data were collected: ward, age, sex, diagnosis, operation name, admission date, discharge date, date of transfused patient’s death, and number of issued blood components. The length of hospital stay was calculated by subtracting the admission date from the discharge date. Data of the total number of outpatients and inpatients over 4 years were also included to track the changes in transfusion rates among the patient groups over time.

To analyze blood component usage by hospital departments and wards, the hospital departments were divided into seven main categories: the intensive care unit (ICU), surgical department, hematology, general medicine, emergency medicine, COVID-19, and others. The COVID-19 category was included in the 2020 analysis to understand the blood component usage for COVID-19 patients. The ICU category included medical, surgical, neonatal, neurological, cardiac, and emergency ICUs. The surgical department category included the outpatient departments that provided surgery or other treatment modalities and inpatient departments, such as orthopedic surgery, urology, spinal surgery, neurosurgery, thoracic surgery, obstetrics and gynecology, general surgery, otolaryngology, ophthalmology, oral surgery, and plastic surgery. Internal medicine was classified under general medicine, and hematology was classified as a separate category, as blood consumption was significantly higher in the hematology department than in the other departments. To analyze the changes in the usage of blood components, total blood was divided into red blood cells (RBCs), platelet concentrates (PCs, a unit of random donor platelets derived from whole blood), plateletpheresis (PP), fresh frozen plasma (FFP), and cryoprecipitate (CRYO).

Since 2009, the Korean medical insurance system implemented a fee-addition system whereby a certain percentage or score is added to the prescribed treatment fee to compensate for differences in resources, financial difficulties, and characteristics of medical institutions [17]. Some of the general surgeries, neurosurgeries, and thoracic surgeries fell under the fee-addition system. Therefore, we classified these surgeries as major surgeries; the remaining surgeries were classified as minor surgeries.

### 2.3. Statistical Analysis

Statistical analyses were performed using SPSS (version 25.0; SPSS, Chicago, IL, USA), R studio 4.1.0 (R studio, Boston, MA, USA), and Prism 8.0 (GraphPad, San Diego, CA, USA). Categorical variables were compared using the chi-square test. For continuous variables, Student’s *t*-test was employed after confirming the normality using the Shapiro–Wilk test. Levene’s test was performed to assess the equality of variance. The Mann–Whitney U test, a nonparametric test, was used for variables with a count of 30 or less. The 95% confidence interval of a proportion is calculated using the hybrid Wilson/Brown method [18]. The effect size in comparison of means was presented by Cohens’ d, and that of proportions by Cohen’s h. Statistical significance was set at *p* < 0.05.

## 3. Results

### 3.1. Overall Blood Components Usage

In 2020, a total of 32,050 blood components were transfused to 2877 patients at our center, representing a 15.8% decrease in transfused units (38,058 units versus 32,050 units) and an 11.8% decrease in transfused patients (3261 patients versus 2877 patients), when compared to the rates in 2019. The total number of inpatients in 2019 was 261,920, and the total number of inpatients in 2020 was 236,915, corresponding to a decrease of 9.5%. The decrease in the number of transfusions was greater than the decrease in the number of patients.

**Figure 1:**
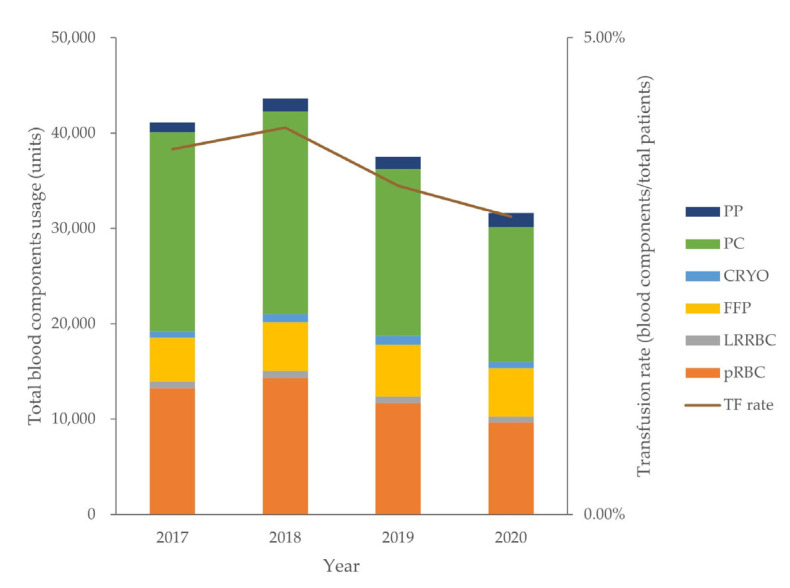
Number of patients visiting the hospital, blood transfusion rate, and overall blood component usage from 2017 to 2020. PP, plateletpheresis; PC, platelet concentrates; CRYO, cryoprecipitate; FFP, fresh frozen plasma; LRFRBC, leuko-reduced filtered red blood cell; pRBC, packed red blood cell; TF rate, transfusion rate of all patients.

The trend towards a decrease in blood transfusions began in 2018. The blood transfusion volume in 2019 decreased by 12.77% compared to 2018. The proportion of patients who received blood transfusions among all hospitalized patients in 2020 decreased significantly compared with that in 2019 (*p* = 0.043). The data of blood components transfused in the last 4 years were analyzed (Figure 1).

The transfusion-to-total-patient ratios were 4.52% in 2019 and 4.06% in 2020. Although the overall number of patients decreased in 2020 compared to that in 2019, the number of transfusions decreased further; hence, the ratios remained stable. PC was the most frequently used blood component, followed by packed RBCs. There were no differences in the order of blood component usage by year.

### 3.2. Blood Usage by Hospital Departments

**Figure 2:**
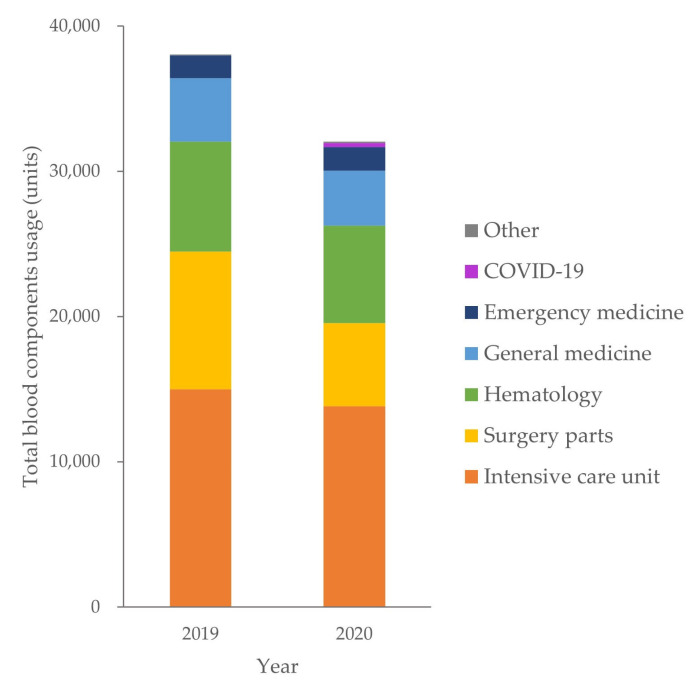
Distribution of blood usage among the clinical departments.

The use of blood components differed by hospital department and ward over the 2 years (Figure 2). The ICU had the highest blood consumption (14,993 units in 2019 and 13,803 units in 2020), followed by the surgery department (9462 units, 5728 units), hematology (7590 units, 6733 units), general medicine (4375 units, 3787 units), emergency medicine (1550 units, 1635 units), and others (88 units, 107 units). The surgery department showed the largest reduction in blood consumption of 39.5% from 2019 to 2020. In 2020, a COVID-19 ward was opened, and its total blood consumption was 257 units for 10 patients with COVID-19.

### 3.3. Blood Usage by Phase of Surgery

As the largest change in blood consumption was observed in the surgery department, we analyzed the blood consumption data according to the phase of surgery: preoperative, intraoperative, and postoperative (Table 1). Transfusion on the day of surgery was classified as intraoperative usage. Of the 7748 units used in the surgery department in 2019, 4261 units (55.0%) were used in the intraoperative phase, and in 2020, of the 5487 units, 3303 units were used in the intraoperative phase (60.2%). Postoperative usage decreased significantly in 2020 (3.87 ± 6.50 units) compared to that in 2019 (7.12 ± 21.71 units) (*p* = 0.047). Next, the surgeries were divided into major and minor surgery, and the changes in usage by blood components were analyzed. There was no significant difference in the postoperative use of total blood components (including RBCs, PC, PP, FFP, and CRYO) between major and minor surgeries between 2019 and 2020. However, analysis by blood component revealed a difference in postoperative RBC usage (Table 2). The postoperative RBC usage in minor surgeries decreased significantly in 2020 (2.18 ± 2.03) compared to that in 2019 (2.88 ± 3.05) (*p* = 0.030).

### 3.4. Prognosis of Postoperatively Transfused Patients

**Figure 3:**
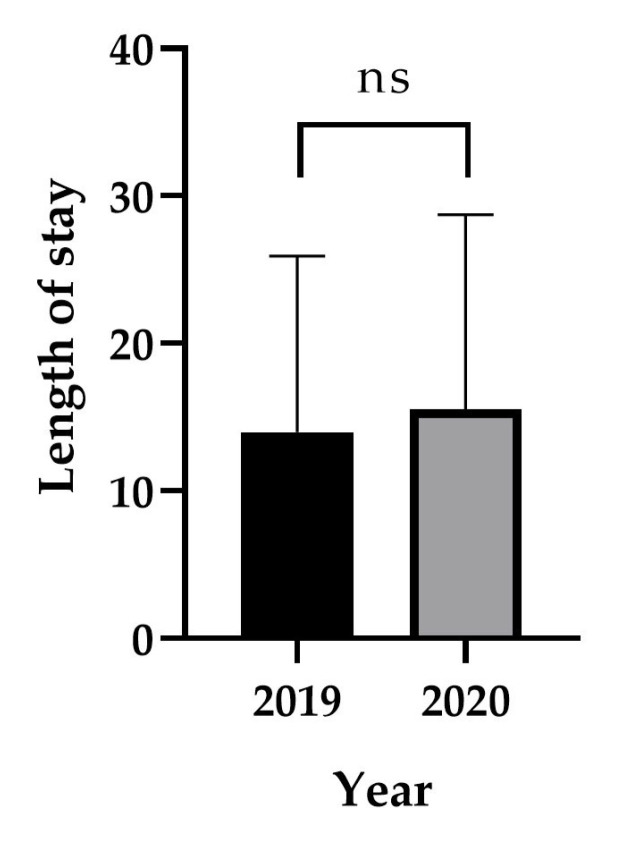
Length of hospital stay of postoperatively transfused patients by year.

The ages of postoperatively transfused patients were analyzed. There was no significant difference in the ages of patients transfused in 2019 (63.97 ± 16.57) and 2020 (62.02 ± 17.61) (*p* = 0.2814). The length of hospital stay and mortality were analyzed to determine whether changes in postoperative transfusion rates affected patient prognosis. The length of hospital stay of the patients who underwent postoperative transfusion in 2019 (*n* = 197) was 13.97 ± 11.95 days, which was not significantly different from that in 2020 (*n* = 167), i.e., 16.44 ± 17.90 days (*p* = 0.118) (Figure 3). In 2019, 9 of 197 postoperatively transfused patients died, and in 2020, 8 of 167 patients died; there was no significant change in the mortality rates between the 2 years (*p* = 0.920). Regarding mortality, among nine patients who died in 2019, two cases died after surgery, and the remaining were found to have died after discharge. In 2020, two out of eight patients died after surgery, and the remaining deaths were not directly related to surgery. In the analysis of mortality and length of hospital stay according to major surgery and minor surgery, there was no significant difference between the groups (Table 3).

### 3.5. Surgery Transfusion Patterns

To determine whether the changes in blood usage from 2019 to 2020 were due to the changes in transfusion rates during surgery, the surgeries performed and the transfusion rates in 2019 and 2020 were analyzed (Table 4). In 2019, of 1,103,612 hospitalized patients, 3105 (0.28%) underwent major surgeries, and in 2020, of 1,025,372 hospitalized patients, 3002 (0.29%) underwent major surgeries. There was no significant change in the proportion of major surgical patients between the 2 years (*p* = 0.1194). The transfusion rate for major surgery was 10.6% in 2019; however, it significantly decreased to 8.8% in 2020 (*p* = 0.017). The transfusion rate for cardiac surgery also significantly reduced (25.4% vs. 16.5%, *p* = 0.005).

For minor surgeries, in 2019, of 1,103,612 hospitalized patients, 9542 (0.86%) underwent minor surgeries, and in 2020, of 1,025,372 hospitalized patients, 8530 (0.83%) underwent minor surgeries. There was a significant reduction in the proportion of minor surgical patients between the 2 years (*p* = 0.0093). The transfusion rate for minor surgery was 7.6% in 2019 and 7.3% in 2020 (*p* = 0.523).

When the type of surgery was categorized by organ (Supplementary Table S1), the RBC transfusion rates for the liver, gall bladder, and pancreas showed a significant decrease in 2020 (*p* = 0.014). The RBC transfusion rate had also decreased for musculoskeletal, cardiovascular, and male genitalia surgeries. Supplementary Table S1 lists the numbers of transfused patients by blood components and the mean numbers of units received by each transfused patient. Overall, RBCs were the most frequently transfused blood components, and PC was the most frequently transfused blood component per transfused patient in both 2019 and 2020.

## 4. Discussion

In this study, we analyzed the blood consumption trends at our hospital during the COVID-19 pandemic and found that the amount of blood consumption had decreased, especially in the surgical department. Moreover, the postoperative transfusion rate had decreased significantly. Regarding blood components, RBC transfusion in minor surgeries showed the highest decline in 2020. Regarding the prognosis of patients who underwent postoperative transfusion, there were no significant changes in the lengths of stay and mortality rates between 2019 and 2020.

A previous study on the use of blood transfusion during the COVID-19 outbreak found that blood consumption was reduced by 50.2% in surgical departments. [13]. The decline in blood consumption could be attributed to the postponement of elective surgeries and increased admissions of patients with COVID-19, which led to a reduction in the demand for routine blood transfusion. [19]. In this study, there was no significant change in the proportion of patients who underwent major surgery in 2019 and 2020. The transfusion rate for major surgery significantly decreased from 10.6% to 8.8% (*p* = 0.017). Furthermore, unlike in the study by Velázquez-Kennedy, [13], only a limited number of patients with COVID-19 were admitted to our hospital; therefore, the amount of blood transfused to these patients did not affect the use of blood products by other patients. The amount of blood transfused during surgery decreased rather than the number of surgeries. It is possible that the COVID-19 pandemic prompted the efficient use of blood products.

Perioperative transfusion can be divided into preoperative, intraoperative, and postoperative, depending on the time of transfusion. Perioperative transfusions aim to enhance the oxygen-carrying capacity, improve hemostasis, and maintain the volume support of cardiac output. [20]. A task force of the American Society of Anesthesiologists developed a consensus on RBC transfusion guidelines for appropriate hemoglobin concentrations before surgical procedures: RBC transfusion is generally necessary when the hemoglobin concentration is less than 7 g/dL. [21,22]. During surgery, intraoperative bleeding can range from gradual oozing to massive rapid arterial blood loss, [23] caused by several factors, including aesthetic agents, patient posture, pneumoperitoneum, and neurological mechanisms. [24]. Subsequently, the hemoglobin concentration may drop rapidly as blood loss increases. Therefore, controlling the number of blood transfusions during surgery is difficult.

In the case of perioperative blood management, there are many possible interventions other than transfusion. Iron deficiency is typically the main cause of anemia. In patients with chronic disease-related anemia, intravenous iron therapy can enhance hemoglobin concentration, functional performance, and quality of life by avoiding these hepcidin-mediated pathways [25,26]. International treatment recommendations advise screening for anemia at least two weeks before surgery in patients who are likely to lose 500 mL or more of blood during surgery, with the advice that anemia be treated with intravenous iron [27,28]. LMW iron dextran, ferumoxytol, ferric carboxymaltose, iron isomaltoside, iron sucrose, and sodium ferric gluconate are exemplary medications for intravenous iron therapy. [29]. 

A recent prospective randomized trial showed that postoperative intravenous infusion of ferric carboxymaltose significantly improved hemoglobin and ferritin concentrations, decreased the transfusion rate, and shortened the length of hospital stay [30]. In a retrospective study, postoperative intravenous administration of iron sucrose to anemic patients accelerated the recovery of hemoglobin concentration [31]. In addition, avoidance of surgical drains and application of cryotherapy to surgical wounds have been utilized to decrease postoperative bleeding [32,33]. 

The Jehovah’s Witnesses, a group of more than 8.5 million individuals worldwide, need special consideration for perioperative blood management because they typically reject blood and blood product transfusions [34]. Once severe anemia is diagnosed, the patients are treated using strategies such as acute normovolemic hemodilution, intraoperative red cell salvage, minimally invasive surgery, and local hemostatic agents [35]. Blood component alternatives such as the experimental hemoglobin-based oxygen carrier (HBOC) are being considered despite the risk of side effects. It has been reported that the Food and Drug Administration has not approved any HBOCs for clinical use to date, and that the only two analytes that remain under discussion are HBOC-201 and PEGylated carboxyhemoglobin bovine (SANGUINATE), which is available from Prolong Pharmaceuticals in South Plainfield, New Jersey. 

In our study, postoperative RBC usage in minor surgeries decreased significantly in 2020 compared to that in 2019. We did not analyze interventions other than blood transfusion in the postoperative period. However, given the “more serious, first serve” concept of prioritization of blood use, the decrease in postoperative transfusions in minor surgeries can be interpreted as the clinician’s response to the insufficient blood supply. As there was no significant difference in the length of hospital stay and mortality of postoperative patients between the 2 years, we considered that the blood products were used efficiently. Due to the lack of blood supply, limited blood products had to be administered based on priority. Minor surgery, in which the disease severity was anticipated to be modest, was the most affected by blood shortage. This indicates that there has been a tendency to overuse blood products previously, and it may be used as evidence to support the idea that reducing inappropriate blood transfusion would not affect the patient’s prognosis. Our findings are also expected to provide basic data for the proper inventory management of blood banks in the lack of blood situation.

Transfusions and transfusion rates were already lower in 2019 than in 2017–2018. Currently, Korea is an aged society (as of September 2022, the proportion of elderly people is 17.8%). The sharp decline in the blood donor population due to changes in the demographic structure is a major problem in the Korean society. In a context of chronic blood inventory shortage, the shortage was exacerbated by the COVID-19 pandemic. On 20 January 2020, Korea had its first COVID-19-confirmed patient, and since then, the number of confirmed patients has increased exponentially. As a result, due to implementation of the social distancing policy and people’s psychological reluctance to go outside, the blood donation rate has sharply decreased, resulting in a significant decrease in domestic blood product reserves. The average amount of blood products in February 2019 was 19,664 ± 3526, and the average amount of blood products in March 2020 was 16,767 ± 2426, showing a 14.7% decrease. The shortage of blood inventory was thought to be worse in medical institutions’ blood centers.

This study had some limitations. First, the need for transfusion is usually determined by laboratory results, such as hemoglobin concentration, platelet count, and prothrombin time, as well as the clinical condition of the patient. However, since the laboratory test results were not analyzed in this study, it was impossible to confirm whether the transfusion rate in 2020 was lower than that in 2019 due to differences in patient’s disease severity or whether the actual transfusion rate was reduced for similar levels of disease severity. Instead, the proportion of patients who underwent major surgery did not change between 2019 and 2020; therefore, it can be indirectly inferred that there was no major difference in disease severity between the 2 years. Second, the relationship between the actual transfusion of blood products and the need for transfusion in each patient was not analyzed in this study. Our hospital’s blood transfusion policy is to transfuse blood products as much as possible to patients who received a prescription for transfusion, assuming that there is no shortage of blood products. RBC preparations are transfused when the hemoglobin level is <7.0 g/dL. Even if the hemoglobin level is >7.0 g/dL and the patient has active bleeding or extracorporeal membrane oxygenation is applied, RBCs are transfused. However, identifying patients who have received blood transfusion prescriptions but have not actually received blood products can provide practical information on the blood shortage situation. Finally, this retrospective study included a single tertiary care institution, and this may limit the applicability of the results to other types of institutions with different blood bank policies and different patient compositions. Multicenter prospective studies can help address the generalizability issues.

## 5. Conclusions

In conclusion, since the COVID-19 pandemic aggravated the blood supply shortage, blood usage decreased for minor surgeries, especially postoperative transfusions; however, patient prognosis was not affected.

## Figures and Tables

**Table 1:** Blood consumption data according to the preoperative, intraoperative, and postoperative periods.

	Year 2019				Year 2020				*p*-Value ^a^	Cohen’s d
	Total Number of Components	Patients	Mean	SD	Total Number of Components	Patients	Mean	SD		
Total operation										
Pre	2085	135	15.44	46.48	1538	104	14.79	26.75	0.898	0.017
Intra	4261	719	5.93	9.28	3303	617	5.35	12.69	0.342	0.052
Post	1402	197	7.12	21.71	646	167	3.87	6.50	0.047 *	0.203
Major operation										
Pre	1130	36	31.39	80.65	432	25	17.28	17.69	0.394	0.242
Intra	1940	239	8.12	11.28	1651	206	8.01	20.50	0.947	0.007
Post	593	53	11.19	25.30	246	32	7.69	12.00	0.465	0.177
Minor operation										
Pre	955	99	9.65	22.43	1106	79	14.00	29.09	0.261	0.167
Intra	2321	480	4.84	7.89	1652	411	4.02	5.16	0.073	0.123
Post	809	144	5.62	20.12	400	135	2.96	3.83	0.122	0.184

^a^ The symbols denote *p*-values * < 0.05; SD, standard deviation.

**Table 2:** Postoperative usage of blood components in major or minor surgeries.

	Year 2019				Year 2020				*p*-Value ^a^	Cohen’s d
	Total Number of Components	Number of Transfused Patients	Mean of Components per Patient	SD of Components per Patient	Total Number of Components	Number of Transfused Patients	Mean of Components per Patient	SD of Components per Patient		
Major operation										
RBC	83	42	1.98	1.16	58	23	2.52	1.65	0.168	0.379
PC	438	16	27.38	37.89	146	5	29.20	18.69	0.919	0.061
PP	9	5	1.80	1.30	26	9	2.89	2.42	0.374	0.561
FFP	63	7	9.00	9.35	16	4	4.00	1.83	0.327	0.742
CRYO	0	0	NA	NA	0	0	NA	NA	NA	NA
Minor operation										
RBC	398	138	2.88	3.05	277	127	2.18	2.03	0.030 *	0.270
PC	331	12	27.58	55.84	82	8	10.25	3.96	0.397	0.438
PP	5	3	1.67	1.15	1	1	1.00	NA	0.667	NA
FFP	73	22	3.32	4.61	40	15	2.67	1.99	0.611	0.183
CRYO	2	1	2.00	NA	0	0	NA	NA	NA	NA

^a^ The symbols denote *p*-values * < 0.05. NA, not applicable; SD, standard deviation; RBC, red blood cell; PC, platelet concentrate; PP, plateletpheresis; FFP, fresh frozen plasma; CRYO, cryoprecipitate.

**Table 3:** Comparison of mortality and length of hospital stay of postoperatively transfused patients in 2019 and 2020.

Variables	Year 2019	Year 2020	*p*-Value	Effect Size ^a^
	(*n* = 197)	(*n* = 167)		
Mortality (cases/total, % ^b^)				
Major surgery	3/53, 5.7 [1.5, 15.4]%	0/32, 0.0 [0.0, 10.7]%	0.171	0.069
Minor surgery	6/144, 4.2 [1.9, 8.8]%	8/135, 5.9 [3.0, 11.3]%	0.501	0.081
Length of hospital stay (Mean ± SD)				
Major surgery	13.06 ± 10.19	13.03 ± 6.77	0.990	0.003
Minor surgery	14.31 ± 12.55	16.1 ± 14.29	0.269	0.133

^a^ Cohen’s h for comparison of proportions and Cohen’s d for means. ^b^ The 95% confidence interval of a proportion is calculated using the hybrid Wilson/Brown method and presented in brackets. SD, standard deviation.

**Table 4:** Surgeries performed in 2019 and 2020 and the corresponding transfusion rates.

	Year 2019				Year 2020				*p*-Value ^b^	Cohen’s h
	Number of Total Patients	Number and % Transfused Patients ^a^		Total Components	Number of Total Patients	Number of Transfused Patients		Total Components		
Major surgery	3105	328	10.6 [9.5,11.7]%	3663	3002	263	8.8 [7.8,9.8]%	2329	0.017 *	0.061
Cardiac surgery	334	85	25.4 [21.1,30.4]%	1098	328	54	16.5 [12.8,20.9]%	741	0.005 **	0.222
Neurosurgery	100	32	32.0 [23.7,41.7]%	199	110	38	34.5 [26.3,43.8]%	194	0.696	0.054
General surgery	2671	211	7.9 [6.9,9.0]%	2366	2564	171	6.7 [5.8,7.7]%	1394	0.087	0.047
Minor surgery	9542	723	7.6 [7.1,8.1]%	4805	8530	625	7.3 [6.8,7.9]%	3158	0.523	0.010

^a^ The 95% confidence interval of a proportion is calculated using the hybrid Wilson/Brown method and presented in brackets. ^b^ The symbols denote *p*-values * < 0.05 and ** < 0.01.

## Data Availability

The data presented in this study are available on reasonable request from the corresponding author. The data are not publicly available due to privacy.

## Supplementary Materials

The following supporting information can be downloaded at: https://www.mdpi.com/article/10.3390/vaccines11030585/s1, Table S1: Blood use during surgery by organ type (for transfused patients only).

## References

[1] Capalbo C., Aceti A., Simmaco M., Bonfini R., Rocco M., Ricci A., Napoli C., Rocco M., Alfonsi V., Teggi A. (2020). The Exponential Phase of the COVID-19 Pandemic in Central Italy: An Integrated Care Pathway. Int. J. Environ. Res. Public Health.

[2] Emanuel E.J., Persad G., Upshur R., Thome B., Parker M., Glickman A., Zhang C., Boyle C., Smith M., Phillips J.P. (2020). Fair Allocation of Scarce Medical Resources in the Time of COVID-19. n. Engl. J. Med..

[3] Truog R.D., Mitchell C., Daley G.Q. (2020). The Toughest Triage—Allocating Ventilators in a Pandemic. n. Engl. J. Med..

[4] Wee L.E., Conceicao E.P., Sim X.Y.J., Aung M.K., Tan K.Y., Wong H.M., Wijaya L., Tan B.H., Ling M.L., Venkatachalam I. (2020). Minimizing Intra-hospital Transmission of COVID-19: The Role of Social Distancing. J. Hosp. Infect..

[5] Lin C.F., Huang Y.H., Cheng C.Y., Wu K.H., Tang K.S., Chiu I.M. (2020). Public Health Interventions for the COVID-19 Pandemic Reduce Respiratory Tract Infection-Related Visits at Pediatric Emergency Departments in Taiwan. Front. Public Health.

[6] Kuitunen I., Artama M., Mäkelä L., Backman K., Heiskanen-Kosma T., Renko M. (2020). Effect of Social Distancing Due to the COVID-19 Pandemic on the Incidence of Viral Respiratory Tract Infections in Children in Finland During Early 2020. Pediatr. Infect. Dis. J..

[7] Savioli G., Ceresa I.F., Novelli V., Ricevuti G., Bressan M.A., Oddone E. (2022). How the Coronavirus Disease 2019 Pandemic Changed the Patterns of Healthcare Utilization by Geriatric Patients and the Crowding: A Call to Action for Effective Solutions to the Access Block. Intern. Emerg. Med..

[8] Rennert-May E., Leal J., Thanh N.X., Lang E., Dowling S., Manns B., Wasylak T., Ronksley P.E. (2021). The Impact of COVID-19 on Hospital Admissions and Emergency Department Visits: A Population-Based Study. PLoS ONE.

[9] Rees E.M., Nightingale E.S., Jafari Y., Waterlow N.R., Clifford S., Pearson B.C.A., Group C.W., Jombart T., Procter S.R., Knight G.M. (2020). COVID-19 Length of Hospital Stay: A Systematic Review and Data Synthesis. BMC Med..

[10] Ou-Yang J., Li S.J., Bei C.H., He B., Chen J.Y., Liang H.Q., Fu Y.S. (2020). Blood Donor Recruitment in Guangzhou, China, During the 2019 Novel Coronavirus (COVID-19) Epidemic. Transfusion.

[11] Gammon R.R., Prichard A.B., Gannett M.S., Yordanov B. (2021). The Effect of COVID-19 on Blood Donation Habits. Transfusion.

[12] Ngo A., Masel D., Cahill C., Blumberg N., Refaai M.A. (2020). Blood Banking and Transfusion Medicine Challenges During the COVID-19 Pandemic. Clin. Lab. Med..

[13] Velázquez-Kennedy K., Luna A., Sánchez-Tornero A., Jiménez-Chillón C., Jiménez-Martín A., Vallés Carboneras A., Tenorio M., García García I., López-Jiménez F.J., Moreno-Jiménez G. (2021). Transfusion Support in COVID-19 Patients: Impact on Hospital Blood Component Supply During the Outbreak. Transfusion.

[14] Kiely P., Hoad V.C., Seed C.R., Gosbell I.B. (2021). Severe Acute Respiratory Syndrome Coronavirus-2: Implications for Blood Safety and Sufficiency. Vox Sang..

[15] Baron D.M., Franchini M., Goobie S.M., Javidroozi M., Klein A.A., Lasocki S., Liumbruno G.M., Muñoz M., Shander A., Spahn D.R. (2020). Patient Blood Management During the COVID-19 Pandemic: A Narrative Review. Anaesthesia.

[16] Shander A., Goobie S.M., Warner M.A., Aapro M., Bisbe E., Perez-Calatayud A.A., Callum J., Cushing M.M., Dyer W.B., Erhard J. (2020). Essential Role of Patient Blood Management in a Pandemic: A Call for Action. Anesth. Analg..

[17] Korean Health Insurance Review and Assessment Service (2017). A Study on the Improvement Plan of the Thoracic Surgery/General Surgery Specialist’s Fee-Addition System. https://www.hira.or.kr/eng/about/05/02/01/index.html.

[18] Brown L.D., Cai T.T., DasGupta A. (2001). Interval Estimation for a Binomial Proportion. Stat. Sci..

[19] WHO Maintaining a Safe and Adequate Blood Supply During the Pandemic Outbreak of Coronavirus Disease (COVID-19). https://www.who.int/publications/i/item/WHO-2019-nCoV-BloodSupply-2021-1.

[20] Ferraris V.A., Ferraris S.P., Saha S.P., Hessel E.A., Haan C.K., Royston B.D., Bridges C.R., Higgins R.S., Despotis G., Society of Thoracic Surgeons Blood Conservation Guideline Task Force (2007). Perioperative Blood Transfusion and Blood Conservation in Cardiac Surgery: The Society of Thoracic Surgeons and the Society of Cardiovascular Anesthesiologists Clinical Practice Guideline. Ann. Thorac. Surg..

[21] American Society of Anesthesiologists Task Force on Perioperative Blood Management (2015). Practice Guidelines for Perioperative Blood Management: An Updated Report by the American Society of Anesthesiologists Task Force on Perioperative Blood Management. Anesthesiology.

[22] Edwards J., Morrison C., Mohiuddin M., Tchatalbachev V., Patel C., Schwickerath V.L., Menitove J.E., Singh G. (2012). Patient Blood Transfusion Management: Discharge Hemoglobin Level as a Surrogate Marker for Red Blood Cell Utilization Appropriateness. Transfusion.

[23] Baker L., Park L., Gilbert R., Martel A., Ahn H., Davies A., McIsaac D.I., Saidenberg E., Tinmouth A., Fergusson D.A. (2019). Guidelines on the Intraoperative Transfusion of Red Blood Cells: A Protocol for Systematic Review. BMJ Open.

[24] Barash P.G., Cullen B.F., Stoelting R.K. (2017). Clinical Anesthesia.

[25] Shepshelovich D., Rozen-Zvi B., Avni T., Gafter U., Gafter-Gvili A. (2016). Intravenous Versus Oral Iron Supplementation for the Treatment of Anemia in CKD: An Updated Systematic Review and Meta-analysis. Am. J. Kidney Dis..

[26] Jankowska E.A., Tkaczyszyn M., Suchocki T., Drozd M., von Haehling S., Doehner W., Banasiak W., Filippatos G., Anker S.D., Ponikowski P. (2016). Effects of Intravenous Iron Therapy in Iron-Deficient Patients with Systolic Heart Failure: A Meta-analysis of Randomized Controlled Trials. Eur. J. Heart Fail..

[27] Muñoz M., Acheson A.G., Auerbach M., Besser M., Habler O., Kehlet H., Liumbruno G.M., Lasocki S., Meybohm P., Rao Baikady R. (2017). International Consensus Statement on the Peri-operative Management of Anaemia and Iron Deficiency. Anaesthesia.

[28] Mueller M.M., Van Remoortel H., Meybohm P., Aranko K., Aubron C., Burger R., Carson J.L., Cichutek K., De Buck E., Devine D. (2019). Patient Blood Management: Recommendations From the 2018 Frankfurt Consensus Conference. JAMA.

[29] Elstrott B., Khan L., Olson S., Raghunathan V., DeLoughery T., Shatzel J.J. (2020). The Role of Iron Repletion in Adult Iron Deficiency Anemia and Other Diseases. Eur. J. Haematol..

[30] Khalafallah A.A., Yan C., Al-Badri R., Robinson E., Kirkby B.E., Ingram E., Gray Z., Khelgi V., Robertson I.K., Kirkby B.P. (2016). Intravenous Ferric Carboxymaltose Versus Standard Care in the Management of Postoperative Anaemia: A Prospective, Open-Label, Randomised Controlled Trial. Lancet Haematol..

[31] Auerbach M., Muñoz M., Macdougall I.C. (2018). Intravenous Iron: Out of Sight, Out of Mind. Lancet Haematol..

[32] Cherian J.J., Kapadia B.H., Issa K., Banerjee S., McInerney V.K., Harwin S.F., Mont M.A. (2013). Preoperative Blood Management Strategies for Total Hip Arthroplasty. Surg. Technol. Int..

[33] Chughtai M., Sodhi N., Jawad M., Newman J.M., Khlopas A., Bhave A., Mont M.A. (2017). Cryotherapy Treatment After Unicompartmental and Total Knee Arthroplasty: A Review. J. Arthroplasty.

[34] Rashid M., Kromah F., Cooper C. (2021). Blood Transfusion and Alternatives in Jehovah’s Witness Patients. Curr. Opin. Anaesthesiol..

[35] Crowe E.P., DeSimone R.A. (2019). Transfusion Support and Alternatives for Jehovah’s Witness Patients. Curr. Opin. Hematol..

