# Supplementary material for: Impact of the COVID-19 Pandemic on the Usage of Blood for Transfusions: A 2-Year Experience from a Tertiary Center in Korea
Source: Vaccines (Basel). 2023 Mar 3;11(3):585. doi: 10.3390/vaccines11030585 (PMC10058496; doi:10.3390/vaccines11030585)
Supplement: Supplementary file 1 [file vaccines-11-00585-s001.zip › vaccines-2158487-supplementary.pdf]

Supplementary Table S1. Blood use during surgery by organ type (for transfused patients only)

	Number of units per transfused patient (N, median [Q1, Q3])																			
	RBC				PC				PP				FFP				CRYO			
	Year 2019		Year 2020		Year 2019		Year 2020		Year 2019		Year 2020		Year 2019		Year 2020		Year 2019		Year 2020	
Section																				
Musculoskeletal	475	3 [2, 4]	376	**2 [2, 3]	35	10 [6.5, 10]	26	10 [8, 18.25]	8	1 [1, 1.5]	11	1 [1, 1.5]	77	3 [1, 4]	48	3 [1.75, 4]	0	NA	0	NA
Liver, GB, pancreas	102	3 [2, 4]	86	*3 [2, 3]	6	10 [10, 13]	5	10 [10, 10]	2	1 [1, 1]	0	NA	14	5 [2, 8.75]	8	2 [2, 4.25]	0	NA	0	NA
GI tract	66	2 [2, 3]	57	2 [2, 4]	12	10 [6, 11]	13	*20 [10, 34]	4	7 [4, 10]	3	1 [1, 2]	18	2.5 [2, 4.75]	12	5 [2, 8.5]	0	NA	0	NA
Spleen and LN	14	2 [1.25, 2]	7	1 [1, 1.5]	10	10 [10, 17.5]	1	8 [8, 8]	2	2 [2, 2]	3	3 [2, 3.5]	0	NA	0	NA	0	NA	0	NA
Circulatory organ	41	7 [6, 12]	33	*5 [3, 6]	12	10 [7.25, 10]	17	10 [6, 13]	27	2 [2, 3]	10	2 [1.25, 2.75]	33	9 [7, 12]	29	**6 [4, 7]	1	4 [4, 4]	4	5 [5, 6.25]
Neural	38	3 [3, 4]	48	3 [2, 4]	6	18 [6.5, 49]	6	6.5 [5, 24.5]	2	1 [1, 1]	3	1 [1, 2.5]	10	6 [3, 11]	5	3 [2, 3]	0	NA	0	NA
Lung	38	3 [2, 3]	24	3.5 [2, 4.25]	2	10 [8.75, 10]	3	10 [7.5, 13]	1	1 [1, 1]	0	NA	16	3 [2, 4.5]	12	2 [2, 3.25]	0	NA	0	NA
Urology	67	3 [2, 4]	50	2 [2, 4.75]	5	10 [8, 14]	9	10 [8, 18]	2	2 [2, 2]	2	1 [1, 1]	9	3 [2, 4]	9	2 [1, 7]	2	4 [3, 5]	0	NA
Female genitalia	71	2 [2, 3]	98	2 [2, 3]	4	15 [13, 61.25]	3	18 [13, 23]	1	3 [3, 3]	1	3 [3, 3]	6	2.5 [2, 3.75]	8	3.5 [2, 4.5]	0	NA	0	NA
Male genitalia	24	3 [2, 4]	20	**3 [2, 4]	2	22 [16, 28]	0	NA	0	NA	0	NA	4	3 [1.75, 4]	1	7 [7, 7]	0	NA	0	NA
Obstetric	6	2.5 [2, 3]	14	2 [1, 2.75]	0	NA	0	NA	0	NA	0	NA	0	NA	2	2.5 [1.75, 3.25]	0	NA	0	NA
Transplantation	5	3 [2, 4]	4	3 [2, 4.5]	2	19 [18.5, 19.5]	0	NA	1	3 [3, 3]	0	NA	2	11.5 [7.25, 15.75]	0	NA	1	3 [3, 3]	0	NA

The symbols correspond to a p-value inferior to *0.05 and **0.01.

RBC, red blood cell; PC, platelet concentrate; PP, plateletpheresis; FFP, fresh frozen plasma; CRYO, cryoprecipitate; GB, gall bladder; GI, gastrointestinal; LN, lymph node
